# Impact of the microbiome on mosquito-borne diseases

**DOI:** 10.1093/procel/pwad021

**Published:** 2023-04-25

**Authors:** Huicheng Shi, Xi Yu, Gong Cheng

**Affiliations:** Tsinghua University-Peking University Joint Center for Life Sciences, School of Medicine, Tsinghua University, Beijing 100084, China; Institute of Infectious Diseases, Shenzhen Bay Laboratory, Shenzhen 518000, China; Tsinghua University-Peking University Joint Center for Life Sciences, School of Medicine, Tsinghua University, Beijing 100084, China; Institute of Infectious Diseases, Shenzhen Bay Laboratory, Shenzhen 518000, China; Tsinghua University-Peking University Joint Center for Life Sciences, School of Medicine, Tsinghua University, Beijing 100084, China; Institute of Infectious Diseases, Shenzhen Bay Laboratory, Shenzhen 518000, China; Department of Parasitology, School of Basic Medical Sciences, Central South University, Changsha 410013, China

**Keywords:** microbiome, mosquito, mosquito-borne viruses, malaria, pathogen transmission

## Abstract

Mosquito-borne diseases present a significant threat to human health, with the possibility of outbreaks of new mosquito-borne diseases always looming. Unfortunately, current measures to combat these diseases such as vaccines and drugs are often either unavailable or ineffective. However, recent studies on microbiomes may reveal promising strategies to fight these diseases. In this review, we examine recent advances in our understanding of the effects of both the mosquito and vertebrate microbiomes on mosquito-borne diseases. We argue that the mosquito microbiome can have direct and indirect impacts on the transmission of these diseases, with mosquito symbiotic microorganisms, particularly *Wolbachia* bacteria, showing potential for controlling mosquito-borne diseases. Moreover, the skin microbiome of vertebrates plays a significant role in mosquito preferences, while the gut microbiome has an impact on the progression of mosquito-borne diseases in humans. As researchers continue to explore the role of microbiomes in mosquito-borne diseases, we highlight some promising future directions for this field. Ultimately, a better understanding of the interplay between mosquitoes, their hosts, pathogens, and the microbiomes of mosquitoes and hosts may hold the key to preventing and controlling mosquito-borne diseases.

## Introduction

Mosquitoes are responsible for transmitting some of the most dangerous pathogens, including filarial nematodes, protozoa (most importantly *Plasmodium*, which causes malaria), and viruses (Gabrieli et al., 2021). *Anopheles* mosquitoes primarily transmit *Plasmodium*, while *Aedes* and *Culex* mosquitoes mainly transmit mosquito-borne viruses, which are predominantly RNA viruses belonging to the *Togaviridae*, *Flaviviridae*, and *Reoviridae* families, as well as the *Bunyaviridae* order, are mainly transmitted by (Harapan et al., 2020; Ma et al., 2021). While some of these pathogens have been known for a long time and pose constant threats, others are emerging and pose a significant risk of causing unprecedented epidemics. Malaria alone caused 247 million cases and 619,000 deaths globally in 2021 (World Malaria Report, 2022). Some of the most epidemiologically important mosquito-borne viruses threaten approximately 3.9 billion people in more than 120 different countries (Shragai et al., 2017; Harapan et al., 2020). In addition, there are emerging novel mosquito-borne pathogens that have caused or may potentially cause epidemics (Weaver and Forrester, 2015; Musso and Gubler, 2016; Weaver et al., 2018; Vue and Tang, 2021). The World Health Organization (WHO) has identified three mosquito-borne diseases as priority diseases in emergency contexts (Jonkmans et al., 2021; *Prioritizing Diseases for Research and Development in Emergency Contexts*), underscoring the danger posed by novel mosquito-borne pathogens.

The threat of mosquito-borne diseases continues to grow, and our current countermeasures are inadequate at best. For example, most mosquito-borne diseases lack effective vaccines (Weaver et al., 2018; Achee et al., 2019), and antimalaria drugs are facing the challenge with resistance (Ashley et al., 2014; Menard and Dondorp, 2017). Additionally, vector control measures such as insecticide-treated nets are losing efficacy due to mosquito resistance (Gatton et al., 2013; Haddi et al., 2017; Gabrieli et al., 2021). There is an urgent need for novel and efficient measures against mosquito-borne diseases. Measures like dietary supplementations that reduce arbovirus prevalence or inhibit mosquito biting have been proposed to meet this need (Zhu et al., 2019; Zhang et al., 2022). It continues to call for a better understanding of mosquito-borne pathogens, especially regarding their interactions with the vector or host microbiomes.

Mosquito-borne pathogens, such as arboviruses and *Plasmodium*, require multistep processes for productive infection in mosquito vectors and vertebrate hosts, offering numerous targets for intervention. Arbovirus infection in mosquitoes begins with viral intake during blood feeding, followed by midgut infection, dissemination to secondary tissues, amplification, crossing the salivary gland barrier, and release into saliva during biting (Hardy et al., 1983; Franz et al., 2015). Upon inoculation into a vertebrate host, the virus needs to find susceptible cells near the biting site, spread from those cells to cause viremia and systematic infection, and may target specific tissues, leading to different pathologies and transmission modes, such as neuropathology, joint pathology, and sexual or vertical transmission (Petersen et al., 2013; Thiberville et al., 2013; Musso and Gubler, 2016). For *Plasmodium*, the mosquito ingestion of male and female gametocytes leads to their fusion, formation of zygotes, and transformation into ookinetes that cross the midgut epithelium, develop into oocysts, and produce sporozoites that move to the circulatory fluid and then the salivary glands (Frischknecht and Matuschewski, 2017). Upon injection into the host dermis, sporozoites enter the blood or lymphatic vessels to reach sites for differentiation (Amino et al., 2006, 2008). In human and other mammalian hosts, sporozoites travel to the liver via the bloodstream, replicate and differentiate in hepatocytes, and produce merozoites that infect red blood cells and undergo asexual replication (White et al., 2014). Some merozoites develop into gametocytes that can be taken up by *Anopheles* mosquitoes. These complex processes offer opportunities for interference by vector or host microbiome.

Mosquito-borne diseases involve complex interactions between vectors and hosts, each harboring complex microbiomes that may impact disease transmission and progression. The microbiota refers to a collection of living microorganisms, including bacteria, archaea, fungi, viruses, and protozoans, within a specific body space or environment. The broader term microbiome can be extended to include molecules such as macromolecules and metabolites produced by the microbiota, as well as other elements like phages, viruses, plasmids, and prions (Berg et al., 2020). The microbiome of an animal can be further divided into tissue-specific microbiomes. Recent research has highlighted the pivotal roles of the microbiome in healthy and pathological phenotypes (Clemente et al., 2012; Perlmutter and Bordenstein, 2020), including development (Sommer and Bäckhed, 2013), immunity (Blander et al., 2017; Gabrieli et al., 2021), and metabolism (Tremaroli and Bäckhed, 2012; Song et al., 2022). In this review, we explore the mechanisms underlying how the microbiome affects mosquito-borne pathogen infection and transmission between mosquito vectors and vertebrate hosts. We also discuss emerging applications of microbiome knowledge in mitigating mosquito-borne diseases.

## The mosquito microbiome

The microbiome of mosquitoes has been found to vary across different tissues, including the midgut (Strand, 2018; Ma et al., 2021), salivary glands (Sharma et al., 2014; Tchioffo et al., 2016; Mancini et al., 2018), reproductive tracts (Ricci et al., 2011; Segata et al., 2016; Tchioffo et al., 2016; Díaz et al., 2021), and cuticle surfaces (Dada et al., 2019, 2021), exhibiting some degree of tissue tropism. These communities of microorganisms in different tissues can have both direct and indirect impacts on mosquito-borne diseases. Direct impacts refer to changes in vectorial competence, which is the ability of mosquitoes to support a productive infection by a pathogen. Indirect impacts are mostly via vectorial capacity, which is the average number of pathogen inoculations by a population of vectors to a host in a specific time period (Garrett-Jones, 1964; Cansado-Utrilla et al., 2021). Vectorial capacity is determined by various factors such as vector biting rate, vector density, vector survival rate, and pathogen extrinsic incubation period, each of which can be influenced by the mosquito microbiome.

### The mosquito gut microbiome

The composition of the mosquito gut microbiome is complex and dynamic, whose discovery dates back to the early 20th century (Hinman, 1930). One early study in 1959 isolated microbes from the midguts of *Culex tarsalis* using a culture method (Chao and Wistreich, 1959), and identified microbes belonging to the phyla Proteobacteria, Firmicutes, Bacteroidetes, and Actinobacteria. Recent works, using a combination of culturing and metagenomic sequencing approaches (Gusmão et al., 2010; Boissière et al., 2012; Osei-Poku et al., 2012; Coon et al., 2014, 2016; Sharma et al., 2014; Dickson et al., 2017), have supported these findings, also identifying fungi such as *Saccharomyces* (yeast) in mosquitoes (Ricci et al., 2011; Coon et al., 2016; Muturi et al., 2016a; Bozic et al., 2017; Tawidian et al., 2021). The composition and diversity of the mosquito gut microbiome are influenced by various factors, including breeding site (Boissière et al., 2012; Tawidian et al., 2021), blood meal (Wang et al., 2011; Muturi et al., 2016a; Calle-Tobón et al., 2021), developmental stages (Dickson et al., 2017), presence of pathogens (Muturi et al., 2016a; Trzebny et al., 2021), and mosquito species (Muturi et al., 2016a, 2016b; Hegde et al., 2018). However, some microbial communities are common to mosquitoes across species or geolocation (Osei-Poku et al., 2012). The intricate relationship between the mosquito gut microbiome, which can exhibit diversity and conservation, and the resulting phenotypes of mosquitoes is currently the subject of intensive investigation (Guégan et al., 2018).

**Figure 1.**
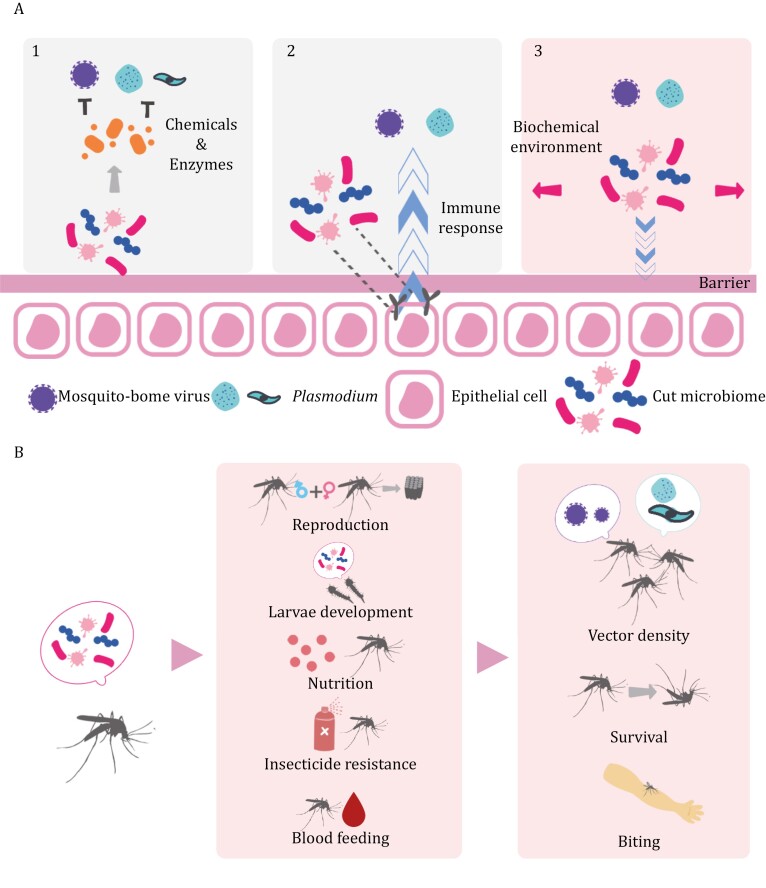
**Direct and indirect effects of the mosquito gut microbiome on the transmission of mosquito-borne diseases.** (A) The mosquito gut microbiome may modulate infection by (1) secreting chemicals and enzymes that inhibit viruses and *Plasmodium* parasites, (2) inducing the immune response, and (3) altering the barrier or biochemical environment in the midgut. (B) The mosquito gut microbiome affects different aspects of mosquito biology, changing the mosquito density, survival, and biting, which are factors modulating disease transmission.

The gut microbiome of mosquitoes directly affects their vectorial competence (Yin et al., 2020; Cansado-Utrilla et al., 2021). Mosquito-borne pathogens are acquired in the gut of mosquitoes through blood feeding and must successfully establish an infection within the mosquitoes to render the mosquitoes competent vectors (Cirimotich et al., 2011; Scolari et al., 2019). In this process, the pathogens are in close proximity to the gut microbiome, which can intervene in their replication. Additionally, the gut microbiome modulates the status of the mosquito gut, thereby changing the environment for pathogen infection. The mechanisms of direct modulation of pathogen infection by the mosquito gut microbiome can be categorized into direct inhibition, immune activation, and environmental modification (Fig. 1A).

The mosquito gut microbiome contains members that produce factors with the ability to directly inhibit mosquito-borne pathogens. *Chromobacterium*, isolated from *Aedes aegypti*, secretes a neutral protease, an aminopeptidase, and two lipases that degrade the protein and lipid membrane of viral particles, disrupting the structure of the dengue virus (DENV) (Saraiva et al., 2018a; Yu et al., 2022). This prevents viral attachment to cells and increases the vulnerability of viral genome to degradation. Furthermore, *Chromobacterium* produces a known anti-malarial agent, romidepsin, which inhibits histone deacetylase (HDAC) (Saraiva et al., 2018b). As a result, incubation with *Chromobacterium* biofilm reduced the activity of *Plasmodium falciparum* and dengue virus serotype 2 (DENV-2) (Ramirez et al., 2014), and oral feeding of antibiotic-treated (aseptic) *A*. *aegypti* with a mixture of human blood, virus, and the culture broth or supernatant of the *Chromobacterium* reduces the infection rate and viral titer of DENV and the Zika virus (ZIKV) in mosquitoes (Yu et al., 2022). The effect of *Chromobacterium* is not specific, as it inhibits the growth of other bacteria from the midgut of mosquitoes when cultured on a plate and shortens the lifespan of mosquitoes after midgut colonization (Ramirez et al., 2014; Yu et al., 2022). However, there are specific effectors like AmLip, a lipase secreted by *Serratia ureilytica* that directly and selectively kills gametocytes (Gao et al., 2021), inhibiting *P*. *falciparum* and *Plasmodium berghei* ookinete. AmLip treatment also leads to the death of *P*. *falciparum* at the asexual stage. Feeding *Anopheles stephensi* and *A*. *gambiae* with *S*. *ureilytica* renders mosquitoes resistant to *P*. *falciparum* without causing apparent changes in mosquito biology. This bacterium is geologically associated with a low number of malaria cases and *Plasmodium vivax* resistance. These findings demonstrate that the mosquito gut microbiome has the potential to directly inactivate pathogens in the mosquito midgut.

The microbiome residing in the mosquito midgut can trigger a basal immune response that plays a crucial role in fighting arboviruses and parasites. This immune response is initiated by the recognition of microbe-associated molecular patterns (MAMPs) by different pattern recognition proteins (PRRs), which then activate the Toll, immune deficiency (IMD), and/or JAK-STAT signaling pathways (Cheng et al., 2016; Pang et al., 2016; Gabrieli et al., 2021). For example, the reintroduction of *Proteus* sp. Prpsp_P or *Paenibacillus* sp. Pnsp_P into aseptic *A*. *aegypti* results in an increase in the transcript abundance of several antimicrobial peptide (AMP) genes, such as Cecropin (*CEC*), Gambicin (*GAM*), and Attacin (*ATT*) (Ramirez et al., 2012). These AMPs are major effectors of the mosquito immune response, and their upregulation occurs locally in the midgut and systematically in the fat body. This reintroduction also decreases the viral titers of DENV-2 in the midgut after the virus is ingested with a blood meal. RNAi silencing of some AMP genes obliterates this effect in the presence of gut bacteria, indicating that these bacteria suppress DENV infection via modulating AMP levels. Similarly, *Chromobacterium* sp. Csp_P induces cecropin *CEC1* promoter activity in a mosquito cell line (Ramirez et al., 2014).

Furthermore, the influence of gut bacteria extends beyond arboviruses and also induces immune responses against other mosquito-borne pathogens. For instance, *Serratia marcescens*, a member of the gut microbiota, activates the mosquito immune system and inhibits the parasite *Plasmodium* (Bai et al., 2019). Transcriptome sequencing analysis of *A. sinensis* midguts colonized by *S*. *marcescens*, isolated from females caught in China, revealed the upregulation of 33 immunity genes including those encoding anti-*Plasmodium* factors thioester-containing protein 1 (*TEP1*), *Anopheles Plasmodium* responsive leucine-rich repeat protein (*APL1A*), leucine-rich repeat protein (*LRRD7*), fibrinogen immunolectin 9 (*FBN9*), *Plasmodium* protective c-type lectin 4 (*CTL4*), and *GAM1*. Additionally, well-known AMP gens Defensin 1 (*DEF1*), *CEC1*, *CEC2*, *GAM1* and most CLIP family genes are also upregulated. The inhibitory effect of *S*. *marcescens* on *Plasmodium* is dependent on the function of transcription factor Relish 2 in the IMD pathway, emphasizing the participation of the immune pathway in the microbiome-mediated immunity against *Plasmodium*. Moreover, after a blood meal in *A*. *stephensi*, expansion of the gut microbiota induces the expression of peptidoglycan recognition protein PGRP-LA, a PRR in the IMD pathway that regulates immune genes (Gao et al., 2020). Knocking down the *PGRP-LA* gene results in the downregulation of 2 AMPs and an anti-*Plasmodium* effector in a panel of ten immune genes and an increase in the infection rate of *P*. *berghei*. However, with the removal of the gut microbiota by antibiotics, *PGRP-LA* knockdown no longer changes the expression of immune genes.

The influence of the microbiome on mosquito-borne pathogens is multifaceted. For instance, in the case of the *A*. *gambiae* mosquitoes, its gut microbiota can have a pro-viral effect on the alphavirus o’nyong-nyong (ONNV) (Carissimo et al., 2015). This is because the midgut bacterial flora elevates the level of the AMP Cecropin 3 (CEC3), which promotes infection of the midgut by ONNV but shows an antiviral function in the hemocoel (Waldock et al., 2012). Thus, the elimination of the gut microbiota with antibiotics appears to hinder ONNV infection of the midgut. On the other hand, when *A*. *aegypti* is provided with a sugar meal of sucrose, glucose, or fructose, they activate several pathways, including siRNA, piRNA, and phenoloxidase (Almire et al., 2021), which provide mosquitoes with resistance to both the Semliki Forest virus (SFV) and ZIKV. However, when the gut microbiota is eliminated with antibiotics, the activation of these pathways is not inhibited but rather amplified. This suggests that the presence of the gut microbiota partially reduces sugar-induced immunity against arboviruses. Clearly, the influence of microbiome-induced mosquito immunity on the infection of mosquito-borne pathogens is intricate and requires further investigation.

The mosquito gut environment is heavily influenced by microbes, which can either promote or inhibit the infection of arboviruses and parasites. The microbiome plays a role in both the physical barrier and biochemical conditions for pathogen infection. The peritrophic matrix (PM), a chitin and glycoprotein layer that protects the mosquito midgut (Hegedus et al., 2009), depends on the microbiome for its post-blood-meal integrity (Rodgers et al., 2017). When the *A*. *stephensi* mosquitoes are treated with antibiotics, the majority of native gut bacteria and PM are absent, resulting in an increase in the number of *P*. *berghei* oocysts (Song et al., 2018). However, the reintroduction of an *Enterobacter* sp. isolated from the mosquito to antibiotic-treated mosquitoes is sufficient to restore PM integrity and reduce *Plasmodium* oocysts to a normal level. Interestingly, the antibiotic treatment also removes bacteria participating in tryptophan metabolism in *A*. *stephensi*, causing the accumulation of 3-hydroxykynurenine (3-HK) which is harmful to the PM (Feng et al., 2022). *Pseudomonas alcaligenes* is a major consumer of 3-HK in the *A*. *stephensi* midgut, and reintroduction of this bacterium is more effective at reducing *P*. *berghei* susceptibility caused by antibiotic treatment than a mutated bacterium lacking the enzyme for converting 3-HK. Conversely, some gut commensal bacteria may impair PM integrity and facilitate infection. *Serratia marcescens* secretes an enhancin-like protein that digests the membrane-bound mucins lining the gut epithelia of mosquitoes (Wu et al., 2019). When introduced orally, either as the bacterium itself or as the secreted Enhancin, it enhances the susceptibility of laboratory-reared and field-caught *A*. *aegypti* and *Aedes albopictus* to different arboviruses. However, an Enhancin-knockout strain does not have the same effect. The mosquito gut microbiome also influences the biochemical facet of the gut environment. The midgut of field-caught *A*. *aegypti* harbors the *Talaromyces* sp. fungus *Tsp_PR*, whose secretome downregulates digestive enzymes and trypsin in the midgut of *A. aegypti* (Angleró-Rodríguez et al., 2017), favoring DENV infection. Colonization of this fungus increases not only the viral load of DENV-2 in *A*. *aegypti* but also the number of *Plasmodium* oocysts in *A*. *gambiae*. Furthermore, the commensal bacterium *Asaia bogorensis* can alkalize the midgut of *A*. *stephensi* when glucose feeding triggers its rapid proliferation (Wang et al., 2021a), promoting both infection and gametogenesis of *P*. *berghei*. Antibiotic treatment can reverse these effects, but recolonization with *A*. *bogorensis* restores the midgut pH and increases the susceptibility to *P*. *berghei*. Therefore, the gut microbiome has a complex impact on the mosquito gut environment, shaping the susceptibility to pathogen infection in diverse ways.

The midgut microbiome has additional indirect links to the transmission of mosquito-borne diseases through vectorial capacity (Fig. 1B). Since the midgut microbiome is involved in various aspects of mosquito biology, it can affect vector biting rate, vector density, and vector survival rate, all of which contribute to vectorial capacity and disease transmission. Evidence supports that mosquito gut microbiome can modulate reproductive behavior and rates, which ultimately impact vector density over time. During mating, the gut microbiome participates in the production of sex pheromones that attract mosquitoes to mate and changes the mating preference of mosquitoes. Microbial metabolites such as sulcatone and acetoin were found to be released at higher amounts by male *A*. *arabiensis* and *A*. *gambiae* during swarming (Mozūraitis et al., 2020), and a mixture with their synthetic analogs attracted *Anopheles* mosquitos of both sexes to increase mating in an experimental setting. Regarding mating preference, apart from some evidence from flies (Sharon et al., 2010, 2011; Englx et al., 2018), a study using transgenic *A*. *stephensi* lines with elevated immune activity suggested that choice in mating is related to the composition of commensal microbes (Pike et al., 2017). Wild-type (WT) male mosquitoes preferentially mated with genetically modified (GM) females and GM males with WT females in the lines with midgut-specific immune enhancement, which reduced the density and altered the composition of midgut microbiota in those transgenic females. This preference was absent after antibiotic treatment that disrupts the gut microbiota. Additionally, the gut microbiome influences the production of offspring in mosquitoes. In the mosquito *Culex pipiens*, the reintroduction of *Bacillus* and *Staphylococcus* to antibiotic-treated mosquitoes rescues or even enhances fecundity (Fouda et al., 2001), indicating that the gut microbiome influences offspring generation. With these different mechanisms, the gut microbiome changes the reproductive outcomes of mosquitoes, which determines the population of mosquitoes, and vectorial capacity increases with the size of a mosquito population.

After the eggs hatch, the gut microbiome during the larval stage also fosters vectorial capacity. The larval microbiome contributes significantly to vectorial capacity by influencing development. Live microbes such as bacteria, yeasts, or an alga in the larval midgut can stimulate hypoxia and promote larval molting (Valzania et al., 2018b). Microbiota supplies riboflavin (vitamin B2) and activates hypoxia-inducible transcription factor (HIF) signaling significant to mosquito growth (Coon et al., 2017; Valzania et al., 2018a; Wang et al., 2021b). Although studies have shown that the need of live bacteria in larval development could be circumvented (Correa et al., 2018; Romoli et al., 2021), it is widely agreed that the presence of gut microbiota promotes normal growth rates and sizes. By modulating the mosquito development, the microbiota alters not only the population density but also the body size of adult mosquitoes, and the body size is correlated with the biting rate, which is the times that a mosquito bites on average in a specific time period, and survival rate, which is the probability of a vector surviving a day (Barreaux et al., 2018; Travanty et al., 2019). Vectorial capacity is positively correlated to all these factors.

The gut microbiome continues to involve in mosquitoes’ vectorial capacity in adulthood. It affects multiple aspects of mosquito biology, including metabolism, insecticide resistance, and host-seeking behavior. The symbionts residing in the mosquito midgut are highly specialized in utilizing the nutrients available in both plant nectar and blood meals (Minard et al., 2013). They provide the mosquitoes with additional metabolic pathways necessary for survival, biting, and reproduction, as in other insects (Rio et al., 2016; Sannino et al., 2018; Wang et al., 2021b). For instance, bacteria and fungi in mosquito midguts actively assimilate fructose, the most abundant component of plant nectar (Guégan et al., 2020). The microbes may participate in the lysis of red blood cells in a blood meal for nutrients, so antibiotic disruption of the gut microbiota reduces the number of eggs laid by mosquitoes (Gaio et al., 2011). These results indicate that the gut microbiome facilitates the digestion of both sugar and blood meals, contributing to mosquito fitness, especially egg development and vector density. The gut microbiome also plays a role in insecticide resistance, with different mosquito strains susceptible or refractory to insecticides harboring different taxa of microbes in their midguts (Wang et al., 2021c). Bacteria that encode insecticide-degrading enzymes are enriched in refractory strains (Dada et al., 2018). Furthermore, the selective modulation of the gut microbiome with various antibiotics differentially changes the susceptibility to different insecticides, including permethrin, deltamethrin, and malathion (Barnard et al., 2019; Gómez-Govea et al., 2022). Microbial-mediated differences in insecticide resistance can affect the mosquito density and, more importantly, their chance of survival. Additionally, the gut microbiome can disrupt the willingness of mosquitoes to take a blood meal, as observed in *A*. *gambiae* with bacterial siderophores (Ganley et al., 2020), *A*. *aegypti* exposed to a strain of *Serratia* (Kozlova et al., 2021), and *A*. *coluzzii* infected by *Chromobacterium violaceum* (Gnambani et al., 2020), potentially reducing their biting frequency. Collectively, the gut microbiome of mosquitoes can influence the number of bites by populations of infected mosquitoes, either increasing or decreasing vectorial capacity, highlighting the significant role that gut microbiota plays in disease transmission.

Recent studies have revealed a bidirectional relationship between the mosquito gut microbiome and mosquito-borne pathogens, with evidence suggesting that pathogens can also affect commensal microbes. For example, both *Aedes triseriatus* and *Aedes japonicus* infected by the La Crosse virus (LACV) have been found to harbor more bacteria taxa and fewer fungal taxa than uninfected mosquitoes (Muturi et al., 2016a). *Anopheles stephensi* fed with a *P*. *vivax* infected blood meal have undetectable bacteria in their midguts for 36 h (Sharma et al., 2020). Similarly, *Elizabethkingia* is more dominant in the midgut of *A*. *albopictus* fed a blood meal with ZIKV than those fed a noninfectious blood meal (Onyango et al., 2021). In females of Colombian *A*. *aegypti* populations, *Bacteroides vulgatus* is significantly enriched in ZIKV-infected mosquitoes, and *Dorea formicigenerans* is elevated in infected mosquitoes (Arévalo-Cortés et al., 2022). Investigating whether arboviruses and parasites can selectively change the composition of the mosquito gut microbiome to facilitate their infection would be crucial to microbiome-based measurements against mosquito-borne diseases.

The modulation of the gut microbiome is being investigated as a strategy to combat mosquito-borne diseases. One promising approach is to vaccinate vertebrate hosts against mosquito gut bacteria, generating antibodies that target specific bacteria and suppress their growth in mosquitoes, thereby inhibiting pathogen infection. For instance, experimental infection of domestic canaries with *Plasmodium relictum*, followed by immunization with Enterobacteriaceae, results in the production of bacteria-specific antibodies (Aželytė et al., 2022). *Culex quinquefasciatus* fed on the infected and immunized birds or birds only immunized have an altered abundance of multiple bacterial taxa in the mosquitoes’ midguts compared to mosquitoes fed on unimmunized counterparts. Immunizing the donor birds with *E*. *coli* O86:B7 reduces both the frequency of malaria infection and the number of oocysts in the midgut of the mosquitoes. Similar experiments in ticks further demonstrate the potential of vaccines against specific components of the arthropod gut microbiome to reduce the transmission of vector-borne diseases (Mateos-Hernández et al., 2020). In addition, the gut microbiome has been explored as a potential tool for the symbiotic control of mosquito-borne diseases, and *Wickerhamomyces anomalus* is a promising candidate in this regard. This yeast is naturally present in the midgut of mosquitoes and has been tested as an antimalaria strategy due to its ability to secrete a killer toxin (KT) against *Plasmodium*, with exo-*β*-1,3-glucanase enzymatic activity (Valzano et al., 2016). Its stable association with *Anopheles* mosquitoes through different stages of the mosquito life history, excellent safety profile, vertical transmissibility, and direct ingestion by both larval and adult mosquitoes as a food source make *W. anomalus* an ideal candidate for the symbiotic control of malaria. In an *in vivo* trial, giving *W. anomalus* as a dietary supplement to *A*. *stephensi* reduced *P*. *berghei* in the midgut by approximately 65%, and the purified KT was shown to be safe and had anti-*Plasmodium* effects in mice (Cappelli et al., 2019). Given that KT can degrade glucans on the surface of various microbes (Walker, 2011), *W. anomalus* may have the potential to control a broad spectrum of mosquito-borne diseases.

### Wolbachia

*Wolbachia*, an intracellularly residing, gram-negative bacteria restricted to ecdysozoans (Landmann, 2019), has drawn considerable attention for its potential in vector control and preventing vector-borne diseases. Present in more than 60% of all insect species (Hilgenboecker et al., 2008), *Wolbachia* has been identified in *Culex*, *Aedes*, and *Anopheles* mosquitoes (Hertig and Wolbach, 1924; Zhou et al., 1998; Shaw et al., 2016; Gomes et al., 2017).

**Figure 2.**
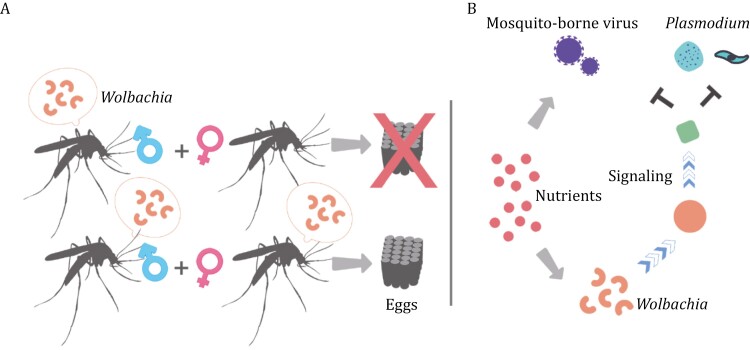
***Wolbachia*-induced cytoplasmic incompatibility and pathogen blocking in mosquitoes.** (A) Only female mosquitoes infected with a compatible strain of *Wolbachia* lay viable eggs after mating with infected males. (B) *Wolbachia* competes for nutrients with and activates immunity against viruses and *Plasmodium*.

Although *Wolbachia* resides in both the somatic and germline tissues of its hosts (Dobson et al., 1999; Osborne et al., 2012), it is most well-known for its reproductive manipulations that enhance its transmission (Werren et al., 2008). Cytoplasmic incompatibility (CI, arrest of development or death of offspring from infected males and uninfected females, as shown in Fig. 2A), as the most common *Wolbachia*-induced phenotype of reproductive distortion, has been observed in mosquitoes (Yen and Barr, 1971). CI is characterized by delayed paternal but not maternal chromatin condensation in embryos, leading to aneuploid or haploid development during the first embryonic mitosis (Callaini et al., 1997). The mechanism behind this phenomenon was hypothesized to be the secretion of a Mod (or toxin) factor(s) by *Wolbachia* into the sperm of infected males, which can cause CI unless the egg is infected by *Wolbachia* producing a corresponding Resc (or antidote) factor(s). The Resc and Mod factors responsible for CI have been identified from two systems (Beckmann et al., 2017; Le Page et al., 2017), with one Mod factor exerting reproductive toxicity via its deubiquitinase activity. If more than one *Wolbachia* strain infects a host, CI can be unidirectional or bidirectional, possibly dependent on the strength of interactions between multiple Mod/Resc factors (Yen and Barr, 1971; Bordenstein and Werren, 2007). In infected females, CI can confer advantages in offspring production, optimizing vertical transmission of *Wolbachia*.

The incompatible insect technique (IIT) is a vector control strategy that was developed based on the *Wolbachia*-induced CI phenomenon (Fig. 2A). In this technique, incompatible males are released into the field in an optimal number, which leads to the elimination of the local population. In the initial trial conducted in 1966, *Culex pipiens fatigans* in a village were eradicated in approximately 3 months (Laven, 1967), which corresponds to 5–6 generations. Currently, IIT is being implemented with the interspecies transfer of *Wolbachia* to *A*. *aegypti* or *A*. *albopictus* (Mains et al., 2019; Zheng et al., 2019; Caputo et al., 2020; Crawford et al., 2020).

Interspecies *Wolbachia* transfer may confer the recipient mosquitoes with protection against various pathogens by modifying the intracellular environment of somatic tissues and inducing signaling (Fig. 2B). The intracellular nature of *Wolbachia* has a profound impact on mosquitoes and mosquito-borne pathogens, as *Wolbachia* relies on the host cell for amino acids and lipids, resulting in an intracellular environment poor in these nutrients, including cholesterol (Caragata et al., 2014; Molloy et al., 2016; Geoghegan et al., 2017). Furthermore, *Wolbachia* occupies and modifies cytoskeletal elements and secretion pathways (Newton et al., 2015; Sheehan et al., 2016; Geoghegan et al., 2017). *Wolbachia* also induces oxidative stress and ER stress in infected cells (Brennan et al., 2008; Pan et al., 2012; Geoghegan et al., 2017), triggering a signaling cascade that activates antiviral or antiparasitic factors (Müller et al., 1997; Kumar et al., 2003; Kambris et al., 2009; Pan et al., 2012; Xu et al., 2013). These changes lead to fitness costs to infected mosquitoes (Min and Benzer, 1997; Rancès et al., 2012). However, due to long-term coexistence with the mosquito, native *Wolbachia* usually causes little stress. *Wolbachia* is expected to stimulate an immune response of modest strength and grow to a low density in its natural host (McGraw et al., 2002; Lindsey et al., 2018; Gabrieli et al., 2021), limiting its pathogen-blocking role. Indeed, in different mosquito species, native *Wolbachia* can be antiviral, not effective, slightly pro-viral, or moderately anti-*Plasmodium* (Table 1, Glaser and Meola, 2010; Mousson et al., 2012; Micieli and Glaser, 2014; Shaw et al., 2016; Skelton et al., 2016; Gomes et al., 2017; Silva et al., 2017). In contrast, novel strains of *Wolbachia* artificially introduced into mosquitoes have shown a potent pathogen-blocking effect (Table 2). For instance, *Wolbachia* from *Drosophila* (*w*Mel, *w*MelPop, or *w*MelPop-CLA) and from *A*. *albopictus* (*w*AlbB) have exhibited inhibitory effects of various degrees on Chikungunya virus (CHIKV), DNEV, ZIKV, yellow fever virus (YFV), and West Nile virus (WNV) in *A*. *aegypti* (Moreira et al., 2009; Bian et al., 2010; van den Hurk et al., 2012; Hussain et al., 2013; Aliota et al., 2016; Dutra et al., 2016). Additionally, *w*Mel-infected *A*. *albopictus* did not develop salivary gland infection by CHIKV (Blagrove et al., 2013). Interspecies-transferred *Wolbachia* also suppressed malaria and filarial parasites in mosquitoes (Kambris et al., 2009; Moreira et al., 2009; Bian et al., 2013). These studies indicate that novel *Wolbachia* infection reduces the infection rate, slows the replication, or prevents the intra-vector transmission to the salivary gland of different pathogens. The ability of foreign *Wolbachia* to impair and even prevent transmission of these pathogens has given rise to a novel population replacement strategy to combat mosquito-borne diseases. This approach involves replacing current mosquito populations with *Wolbachia*-infected ones that are resistant to the target pathogens. The efficacy of this strategy has been tested in a semifield setting and then deployed in multiple locations globally, using *w*Mel- or *w*MelPop-infected *A*. *aegypti* lines (Hoffmann et al., 2011, 2014; Walker et al., 2011; Nguyen et al., 2015; Gesto et al., 2021a, 2021b; Hien et al., 2022). This led to not only the establishment of *Wolbachia* in *A*. *aegypti* populations under most circumstances but also a reduction in the incidence of DENV, ZIKV, and YFV (Ryan et al., 2020; Pinto et al., 2021). For example, *w*Mel-infected *A*. *aegypti* were deployed in a recent geographically cluster-randomized trial in Yogyakarta, Indonesia, between March and December 2017, and a 77% reduction in dengue incidence with an 86% reduction in dengue hospitalizations regardless of DENV serotypes was observed from January 2018 to March 2020 (Utarini et al., 2021). In summary, both the reproduction-manipulating and pathogen-blocking functions of *Wolbachia* in mosquitoes have promising applications for controlling mosquito-borne diseases.

**Table 1.** Effect of native *Wolbachia* on mosquito-borne pathogens.

Mosquito species	Pathogen(s)	Effect	References
*Aedes albopictus*	DENV-2	Did not reduce head infection rate	Bian et al. (2010)
*Aedes albopictus*	CHIKV	Did not significantly change viral replication, dissemination or transmission	Mousson et al. (2010)
*Culex quinquefasciatus*	WNV	Slightly reduced viral replication, significantly reduced dissemination and transmission	Glaser and Meola (2010)
*Aedes albopictus*	DENV-2	Did not affect viral replication, but limited dissemination and transmission	Mousson et al. (2012)
*A. fluviatilis*	*P. gallinaceum*	Had no effect on or increased the intensity of oocysts in the midgut	Baton et al. (2013)
*Culex quinquefasciatus* *Culex pipiens*	WNV	Showed no effect due to low density	Micieli and Glaser (2014)
*Culex pipiens*	*P. relictum*	Increased the oocyst prevalence and density in midgut	Zélé et al. (2014)
*A. notoscriptus*	DENV-2	No effect on the midgut infection rate or titer	Skelton et al. (2016)
*A. coluzzii*	*Plasmodium*	Was negatively correlated with the presence of *Plasmodium*	Shaw et al. (2016)
*A. fluviatilis*	DENV-2 DENV-3	Did not affect the infection rate or viral titer	Silva et al. (2017)
*A. gambiae* *A. coluzzii*	*P. falciparum*	Was negatively correlated with the presence and the intensity of *P. falciparum* in field-caught mosquitoes	Gomes et al. (2017)

**Table 2.** Effect of interspecies-transferred *Wolbachia* on mosquito-borne pathogens.

Mosquito species	*Wolbachia* strain	Pathogen(s)	Effect	References
*Aedes aegypti*	*w*MelPop	*Brugia pahangi*	Significantly reduced the mean numbers of third larval stage microfilariae and the prevalence of infection	Kambris et al. (2009)
*Aedes aegypti*	*w*MelPop-CLA	DENV-2 CHIKV *P. gallinaceum*	Almost blocked oral infection of DENV-2, reduced the DENV-2 titer and prevented DENV-2 from disseminated into saliva in intrathoracically injection, reduced the titer and blocked viral dissemination of CHIKV after oral infection, and reduced the prevalence and intensity of *Plasmodium* oocysts	Moreira et al. (2009)
*Aedes aegypti*	*w*AlbB	DENV-2	Reduced viral replication, dissemination and transmission	Bian et al. (2010)
*A. gambiae*	*w*MelPop	*P. berghei*	Decreased the intensity of *Plasmodium*	Kambris et al. (2010)
*A. gambiae*	*w*MelPop *w*AlbB	*P. falciparum*	Both reduced the intensity of *Plasmodium* oocysts	Hughes et al. (2011)
*Aedes aegypti*	*w*Mel *w*MelPop	DENV-2	Reduced viral replication and blocked dissemination and transmission	Walker et al. (2011)
*A. polynesiensis*	*w*AlbB	*Brugia pahangi*	Reduced the load of infectious third stage worm and mosquito survivorship	Andrews et al. (2012)
*Aedes aegypti*	*w*Mel	CHIKV YFV	Reduced the prevalence and dissemination of CHIKV after oral infection, lowered the prevalence of YFV in intrathoracic inoculation with a low dose, and reduced the body and head titer of YFV after inoculation with both low and high doses	van den Hurk et al. (2012)
*Aedes albopictus*	*w*Mel	DENV-2	Prevented the presence of virus in saliva	Blagrove et al. (2012)
*A. gambiae*	*w*AlbB *w*MelPop	*P. berghei*	The prevalence of *Plasmodium* was not affected. *w*AlbB increased midgut oocyst density but *w*MelPop decreased the density	Hughes et al. (2012)
*Aedes albopictus*	*w*Mel	CHIKV	Prevented the presence of virus in saliva	Blagrove et al. (2013)
*Aedes aegypti*	*w*Mel *w*MelPop	WNV	*w*Mel slowed viral replication after intrathoracic injection, while *w*MelPop reduced the prevalence and titer and stopped virus transmission	Hussain et al. (2013)
*A. stephensi*	*w*AlbB	*P. falciparum*	Reduced the prevalence and intensity of midgut oocysts and salivary gland sporozoites	Bian et al. (2013)
*Culex tarsalis*	*w*AlbB	WNV	Increased the prevalence 7 days post-feeding but did not cause significant changes in viral titer or dissemination	Dodson et al. (2014)
*Aedes aegypti*	*w*Mel + *w*AlbB	DENV	DENV-2 prevalence after intrathoracic injections in *w*Mel + *w*AlbB mosquitoes were lower than those in the *w*Mel line and *w*AlbB line. After feeding on blood from viremic dengue patients, *w*Mel*w*AlbB had stronger inhibitory effect on salivary viral prevalence and titer than *w*Mel, but had similar effect on the infection of abdomen	Joubert et al. (2016)
*Aedes aegypti*	*w*Mel	ZIKV	Decreased ZIKV prevalence and dissemination after oral infection, reduced viral prevalence and titer in saliva	Dutra et al. (2016)
*Aedes aegypti*	*w*Mel	ZIKV	Reduced the prevalence and dissemination of ZIKV, prevented its transmission	Aliota et al. (2016)
*Aedes aegypti*	*w*AlbB	DENV-2 WNV	Replication and transmission of viruses were inhibited in both stable and transient models of *Wolbachia* infection. Stable infection of *w*AlbB had stronger pathogen-blocking effect and prevented infectious virus from entering saliva	Joubert and O’Neill (2017)
*Aedes aegypti*	*w*Au	SFV DENV-2 ZIKV	Reduced the viral titer of SFV, the prevalence and dissemination of DENV-2 to a degree lower than *w*AlbB and *w*Mel, completely eliminated ZIKV replication	Ant et al. (2018)
*Aedes aegypti*	*w*Mel	Mayaro virus	Reduced the prevalence and titer of the virus and stopped transmission	Pereira et al. (2018)
*Aedes aegypti*	*w*Mel	ZIKV/DENV-1 ZIKV/DENV-3	Inhibited the mono-infection of ZIKV, DENV-1 and DENV-3, reduced the prevalence of infection and titers of both viruses in the co-infection	Caragata et al. (2019)
*Aedes aegypti*	*w*AlbA	ZIKV	Reduced the viral prevalence and dissemination and prevented viral transmission in oral infection	Chouin-Carneiro et al. (2020)

### The microbiome in other tissues and the virome

While much research has been focused on the mosquito gut microbiome and the effects of *Wolbachia*, the potential direct and indirect effects of other symbiotic microbes on mosquito-borne disease transmission remain largely unexplored. Furthermore, recent interest has been growing regarding the interactions between the viral communities that infect mosquitoes and the pathogens they transmit. It is important to investigate these understudied areas in order to gain a more complete understanding of the complex interactions between mosquitoes, their microbiome, and the pathogens they transmit.

Although microbial communities have been identified in mosquito tissues other than the midgut, such as the reproductive tissue and salivary gland (Sharma et al., 2014; Tchioffo et al., 2016; Mancini et al., 2018), direct evidence supporting their impact on disease transmission is lacking (Onyango et al., 2020). Nevertheless, it is plausible that these microbiomes could activate an immune response similar to the gut microbiome, potentially influencing the transmission of mosquito-borne pathogens. Additionally, the microbiome of reproductive tissue may affect vectorial capacity via vector density. Research on other arthropods has shown that microbiota can damage reproductive tissues, leading to reduced reproductive rates and impaired offspring development (Perlmutter and Bordenstein, 2020). These factors can impact the size of a mosquito population over time. Despite this, there is no direct evidence of the microbiome in mosquito reproductive tissues affecting mosquito population density. The salivary gland, like the midgut, is also a site where pathogens replicate in the presence of abundant microbial communities. Commensal microbes in the salivary glands of mosquitoes may interact with pathogens there in a similar way to the gut microbiome, potentially interfering with mosquito-borne diseases. For instance, bacteria such as *Asaia* have been identified in mosquito salivary glands (Favia et al., 2007; Damiani et al., 2010), and their replication has been correlated with decreases in *Plasmodium* (Capone et al., 2013). However, it is not yet known whether *Asaia* interacts with malaria sporozoites that accumulate in the salivary glands of *Anopheles* mosquitoes (Frischknecht and Matuschewski, 2017). Unlike microbes in other tissues, the salivary gland microbiome may affect mosquito-borne diseases by modulating the host immune system. Salivary factors can adjust the immune responses of vertebrate hosts (Mellink and Vos, 1977; Demeure et al., 2005; Depinay et al., 2006; Gavor et al., 2022; Martin-Martin et al., 2022), and some arboviruses exploit these mechanisms to evade immune destruction and find host immune cells as their initial replication sites (Schneider et al., 2010; Styer et al., 2011; Briant et al., 2014; Conway et al., 2014; Schmid et al., 2016; Sun et al., 2020). Therefore, it is possible that mosquito commensal microbes participate in the recruitment of host innate immune cells and the initiation of infection. Interestingly, it has been discovered that sandfly gut microbes can be egested into the host during biting, promoting the disease caused by *Leishmania* (Dey et al., 2018). To be specific, the mosquito symbiotic bacteria injected into mammalian hosts have been found to upregulate IL-1β, enhance the capture and protection of *Leishmania* by neutrophils, and heighten the severity of leishmaniasis. This finding suggests that mosquitoes may also egest their commensal microbes when feeding on vertebrate hosts, which could modulate the host immune response in concert with salivary proteins to facilitate the establishment of mosquito-borne diseases. Nonetheless, research on the effects of microbiomes in the reproductive tract and salivary gland on mosquito-borne diseases is still limited, probably due to the challenges of manipulating these microbiomes without affecting the gut microbiome.

Recent advances in metatranscriptomics have uncovered the presence of insect-specific viruses (ISVs) in mosquitoes, which cannot infect vertebrates (de Almeida et al., 2021; Ren et al., 2021; Gómez et al., 2022). These viruses, collectively known as the virome, are typically nonpathogenic to mosquitoes but have implications for mosquito-borne diseases. Mosquito ISVs are mostly RNA viruses belonging to *Flaviviridae*, *Bunyaviridae*, *Rhabdoviridae*, *Reoviridae*, and *Togaviridae* families (Atoni et al., 2019; de Almeida et al., 2021), and some of them are phylogenetically regarded as ancestral to some arboviruses. ISVs, which are highly prevalent in mosquitoes, have been shown to modulate arbovirus infection. For instance, the cell-fusing agent virus (CFAV), one of the first characterized ISVs, was found to elevate the expression of the known pro-viral factor ribonuclease kappa in *A*. *aegypti* Aag2 cells, thereby enhancing DENV infection (Zhang et al., 2017). However, *in vivo*, mosquito ISVs tend to compete with and suppress arboviruses. For example, the Nhumirim virus (NHUV), an insect-specific flavivirus, has been found to reduce the titer and transmissibility of WNV in *C*. *quinquefasciatus* but not in *C*. *pipiens* (Goenaga et al., 2015). It has also been found to reduce the ZIKV infection rate and transmissibility in *A*. *aegypti* (Romo et al., 2018). In contrary to the *in vitro* study, pre-exposure of *A*. *aegypti* mosquitoes to CFAV leads to a reduction in both DENV-1 and ZIKV titers in mosquito heads (Baidaliuk et al., 2019). Similarly, in *C*. *quinquefasciatus* fed with WNV, mosquitoes with an insect-specific sobemo-like virus have no infectious WNV in their heads (Shi et al., 2022). Additionally, certain members of the mosquito virome may directly infect *Plasmodium* in malaria (Bird et al., 1972), though the consequence of such an interaction is unknown. Overall, the interaction between ISVs and pathogens is a newly emerging area that has high potential in disease control.

## The vertebrate host microbiome

### The skin microbiome and transmission of mosquito-borne diseases

The skin microbiome of vertebrate hosts impacts the transmission of mosquito-borne diseases by producing volatiles that either attract or repel mosquitoes, influencing their host-seeking behavior. These volatile compounds such as short- and medium-chain fatty acids, fatty acid derivatives, and short-chain amino acids are produced by skin commensal microorganisms and contribute to the overall body odor (James et al., 2004; Verhulst et al., 2010; Duffy and Morrin, 2019). Mosquitoes are triggered by odors (Dekker et al., 2005; Spitzen et al., 2008), and the successful search for vertebrate hosts is critical for the life cycles of mosquito-borne pathogens. Therefore, the attractiveness of skin microbial metabolites has an impact on the transmission of mosquito-borne diseases.

**Figure 3.**
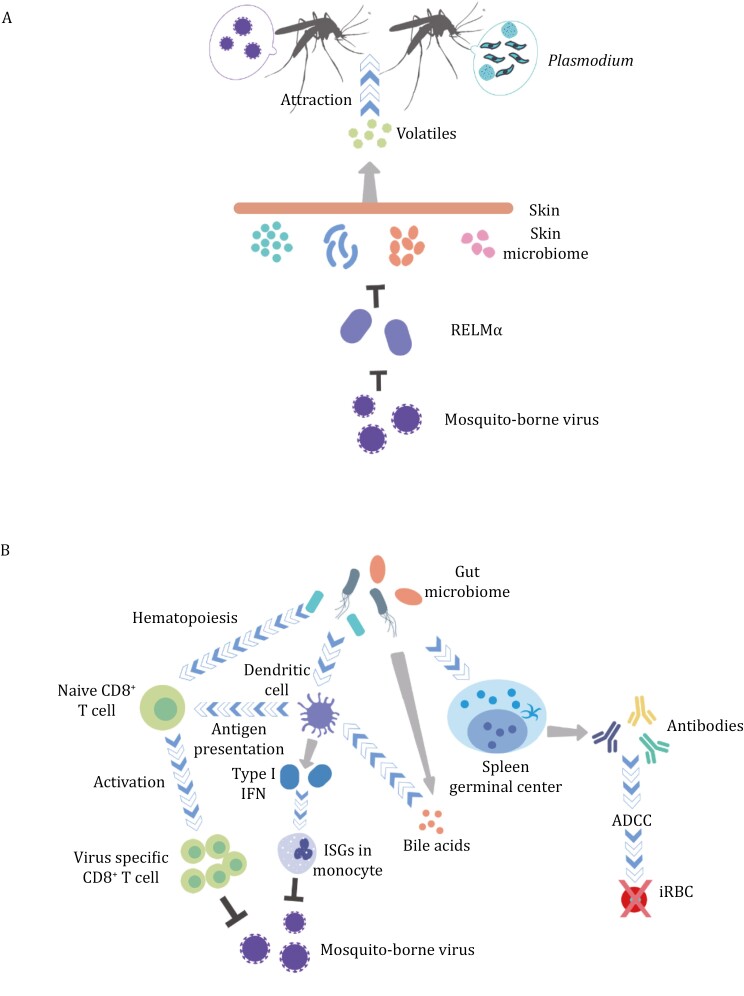
**Interference by the microbiome of vertebrate hosts with mosquito-borne diseases.** (A) The skin microbiome contributes to the attraction to disease-transmitting mosquitoes, and some arboviruses regulate the skin microbiome. (B) The gut microbiome facilitates the immune responses against mosquito-borne viruses and *Plasmodium*.

Accumulating evidence suggests that the host skin microbiome affects mosquito behaviors, and pathogens transmitted by mosquitoes take advantage of this effect (Fig. 3A). The idea has been around for a long time: as early as 1968, Schreck and James demonstrated that culture broths of *Bacillus cereus* collected from a human’s arm were attractive to female *A*. *aegypti* (Schreck and James, 1968). Subsequent studies showed that not only the abundance and diversity of bacteria on human volunteers’ skin (Verhulst et al., 2011), but also the animals from which skin bacteria were collected, could affect the attractiveness to *Anopheles* mosquitoes (Busula et al., 2017). These findings imply that the skin microbiome can influence both intraspecies and interspecies host preferences of mosquitoes. The plasticity of mosquito host preferences is critical in the transmission of mosquito-borne diseases as many pathogens have wildlife reservoirs (Chen and Vasilakis, 2011; Weaver and Forrester, 2015; Figueiredo, 2019). Mosquitoes act as bridges between wildlife and humans, connecting the sylvatic cycles of pathogens to the cycles between humans and mosquitoes. The degree of host preference plasticity also plays a role in determining the likelihood of new mosquito-borne diseases spilling over to humans. Recent research has shown that pathogens such as DENV and ZIKV can also manipulate the skin microbiota of mammalian hosts in favor of their own transmission (Zhang et al., 2022). For example, DENV-2 or ZIKV infection can upregulate skin commensal bacteria, some of which produce acetophenone, a compound that attracts more mosquitoes to the infected host, thereby facilitates the dissemination of these arboviruses (Fig. 3A).

Understanding the role of the skin microbiome in mosquito-borne disease transmission has led to innovative ideas for disease control. One approach involves modifying traps to utilize bacterial volatiles as attractants. (Spitzen et al., 2008; Verhulst et al., 2011). Another strategy is to regulate the host skin microbiome to block disease transmission. For example, Zhang et al. (2022) demonstrated that inducing resistin-like molecule-α (RELMα), an epidermal antimicrobial protein, in DENV-2- or ZIKV-infected mice by dietary administration of a vitamin A derivative inhibited the expansion of *Bacillus* spp. that produce the mosquito-attracting volatile acetophenone. As a result, fewer mosquitoes successively fed on the mice and acquired the viruses. These findings highlight the potential of leveraging knowledge about the attractiveness of the human skin microbiome to mosquitoes to augment current measures for controlling mosquito-borne diseases.

### The microbiome and progression of mosquito-borne diseases

The human host’s complex microbiome has a significant impact on the progression of mosquito-borne diseases, and the magnitude and duration of pathogens in the blood can affect disease transmission in subsequent rounds. The gastrointestinal (GI) tract microbiome has been the most extensively studied in this regard, and it exhibits intricate interactions with mosquito-borne pathogens, typically involving host immune responses.

Human immune responses to mosquito-borne pathogens, including arboviruses and *Plasmodium*, rely on both innate immunity and adaptive immunity. Pathogen recognition receptors, such as Toll-like receptors (TLRs) 3 and 7, detect arboviruses by sensing their RNAs, which then activate downstream transcription factors that induce interferon (*IFN*) expression (Nelemans and Kikkert, 2019; Pierson and Diamond, 2020). IFNs function through IFN-stimulated genes (ISGs) in the early stage of viral infection. Activities of ISGs differ depending on the combinations of genes and viruses (Schoggins, 2014; Nelemans and Kikkert, 2019). The adaptive immunity that resolves arboviral infection necessitates the humoral and cellular arms of the immunity. Typically, virus-neutralizing antibodies play a critical role in controlling infection (Crill and Roehrig, 2001; Diamond et al., 2003; Fernandez et al., 2018). However, there are instances of antibody-dependent enhancement (ADE) in DENV infection, where low titers of antibodies or antibodies against DENV of a different serotype mediate viral entry exploiting the Fc-receptor, leading to more severe disease (da Silva Voorham et al., 2012; Katzelnick et al., 2017; St. John and Rathore, 2019). T-cell responses in arboviral infection are also double-edged swords. CD4^+^ T cells can clear the virus by producing antiviral cytokines and regulating antibody responses and CD8^+^ T cells (Sitati and Diamond, 2006; Yauch et al., 2010; Aberle et al., 2018), but skewed production of cytokines, usually by memory cells, can have destructive consequences (Beaumier and Rothman, 2009). CD8^+^ T cells participate in viral clearance by lysing infected cells and are also capable of producing cytokines (Yauch et al., 2009; Elong Ngono et al., 2017). However, the CD8^+^ T-cell response can be pathologic in some circumstances (Jurado et al., 2018). In malaria, both innate and adaptive immunities are both involved in the defense as well. Sensing of parasitic pathogen-associated molecular patterns (PAMPs) leads to the production of IFNs and recruitment of natural killer (NK) cells, which reduce the parasite burden in both the liver and blood stage (Goodier et al., 2020; Pohl and Cockburn, 2022). During the liver stage, CD8^+^ T-cell responses and cytokines such as IL-10, IL-12p70, IL-6, and TNF exhibit a strong antimalaria effect (Holz et al., 2016; Sato et al., 2019). In the blood stage, antibodies play a major protective role by inducing antibody-dependent cellular cytotoxicity (ADCC) against infected red blood cells (iRBCs) (Cohen et al., 1961). The breadth of the antibody response to diverse malaria antigens increases over repeated exposure and correlates with alleviated clinical symptoms (Osier et al., 2008; Weiss et al., 2010; Nogaro et al., 2011).

As the human gut microbiome plays different roles in human activities, it can manipulate the processes of mosquito-borne diseases, partially by modulating the immune response. One way the gut microbiome can affect pathogens from mosquito bites is by promoting healthy skin and modulating cutaneous immunity (Levkovich et al., 2013; Salem et al., 2018). Another possible mechanism is distal priming of immunity (Fig. 3B) through means including the secretion of DNA-containing membrane vesicles that activate the cGAS-STING-IFN-I axis to induce a systematic antiviral type I IFN response (Erttmann et al., 2022). The impact of the gut microbiome on mosquito-borne viruses has been studied in mouse models. Oral antibiotic treatment in these models has been shown to increase the death rate after WNV, DENV, or ZIKV infection and increase CHIKV titer and dissemination (Thackray et al., 2018; Winkler et al., 2020). The increased susceptibility to flaviviruses is thought to be due to a reduction in hematopoiesis in the bone marrow and a decrease in the number of antigen-presenting dendritic cells in the spleen, resulting in an impaired CD8^+^ T-cell response (Fig. 3B). Treatment with different combinations of antibiotics has also demonstrated that the composition of the gut microbiota correlates with susceptibility to WNV. Mice depleted of gut microbiota or germ-free mice challenged by CHIKV have lower type I IFN production by plasmacytoid dendritic cells, leading to circulating monocytes that activate the expression of fewer ISGs and become permissive to CHIKV (Fig. 3B), thereby facilitating the systematic spread of CHIKV. However, immunity against CHIKV in normal mice is TLR7 and MyD88 dependent and can be restored by recolonization of a single *Clostridial* bacterium. In addition to mosquito-borne viruses, the gut microbiome also plays a role in malaria infection (Fig. 3B). In mice of the same genetic background but with different microbiota compositions, the severity of malaria infection varied, and this difference correlated with the magnitude of humoral immune responses (Villarino et al., 2016). The trend in susceptibility can be regenerated in germ-free mice receiving microbiota transplants from the original mice. Moreover, a study in children confirmed the correlation between gut microbiota composition and malaria outcomes (Mandal et al., 2021). The effect of gut commensal bacteria on the humoral immune response and *Plasmodium* burden was found to be dynamic, and antibiotic treatment in the mouse model was shown to enhance immunity to *Plasmodium*.

The impact of the human microbiome on mosquito-borne pathogens is not limited to the gut; certain tissues can also be affected. For example, in an *ex vivo* vaginal mucosal infection model consisting of vaginal epithelial cells (VECs), colonization of different clinical vaginal microbiome (VMB) samples affected ZIKV infection (Amerson-Brown et al., 2019). VMB samples that inhibited ZIKV had higher levels of IL-22 and platelet-derived growth factor (PDGF)-BB, while those that promoted ZIKV had higher RANTES (regulated upon activation, normal T-cell expressed and presumably secreted) and lower IL-1 levels, with different dominant bacterial species associated with each effect. Importantly, the study found that the effect on ZIKV infection was independent of TLRs. This highlights the importance of incorporating microbiome analysis into *ex vivo* models for mosquito-borne diseases to better reflect *in vivo* conditions.

The microbiome can be a useful prognostic tool in clinical settings when assessing patients at the early stages of mosquito-borne infections. As previously mentioned, the composition of the host microbiome affects the outcome of infection. One study conducted in Mali showed that the microbial makeup of stool samples before a malaria transmission season was associated with the prospective risk of *P*. *falciparum* infection (Yooseph et al., 2015). Furthermore, pathogens carried by mosquitoes can also affect the gut microbiota (Corrêa et al., 2021). For instance, patients infected with DENV who developed critical conditions displayed a higher abundance of Proteobacteria, a higher prevalence of endotoxin and (1→3)-β-d-glucan (BG) produced by microbes in serum as well as signs of gut microbiota and microbial product leaked into the bloodstream at enrollment (Chancharoenthana et al., 2022). Conversely, patients who only experienced febrile conditions did not exhibit these features. Consequently, monitoring the microbiome of patients can aid in the prevention and treatment of mosquito-borne diseases.

Efforts have been made to translate our understanding of the microbiome’s regulation of mosquito-borne diseases into actionable strategies to combat these illnesses. One such approach involved testing whether vaccination against Galα1-3Galβ1-4GlcNAc-R (α-gal) glycan, a sugar molecule that exists in both microbes and *Plasmodium*, could provide protection against malaria (Yilmaz et al., 2014). Humans lack the gene required to produce α-gal but can produce antibodies to it. Studies found that titers of anti-α-gal IgM correlated with exposure to malaria and that this type of antibody may play a protective role in malaria infection. In mice lacking α-gal, colonization with a gut pathobiont stimulated an IgM response. Immunization with α-gal in these mice generated IgM and IgG responses that reduced the *Plasmodium* infection rate. If similar responses could be elicited in humans, vaccination with a commensal microbe could produce antibodies against *Plasmodium*. However, caution is warranted in areas where both malaria and dengue fever are prevalent, as a recent study found that high levels of anti-α-gal IgG and IgG1 were associated with severe dengue fever (Olajiga et al., 2022). Therefore, the application of vaccination against α-gal requires further investigation to fully understand the role of α-gal antibodies in arboviral infection.

## Perspectives

The interaction between the mosquito microbiome and mosquito-borne pathogens has generated significant interest, and with the advancements in high-throughput DNA sequencing technologies, there has been a growing elucidation of the various components of the mosquito microbiome and their roles in infection or transmission of pathogens. The symbiotic control strategy has yielded some success in mitigating mosquito-borne diseases. Similarly, the skin microbiota’s impact on mosquito preference and the human microbiota’s effect on mosquito-borne diseases have been discovered over the past decade, but the conclusions are still at an early stage. Several questions regarding the mosquito microbiome remain to be answered, and some challenges still need to be addressed to control mosquito-borne diseases through microbial means.

One key question that needs to be addressed is why some microorganisms show different effects on a type of pathogen in different studies. For instance, a bacterium that was found to have anti-malarial properties in one context might promote malaria in another. To fully understand the role of a specific microbiome component, it is necessary to investigate whether its effect is dependent on mosquito species or tissue type. Furthermore, it is important to determine whether this microorganism is effective against one specific pathogen or a broad range of viruses or malaria parasites. Another crucial factor to consider is that the composition of the microbiome can vary between studies, potentially masking or reversing the influence of a particular component. Identifying microorganisms that consistently show either pro- or anti-pathogen effects, regardless of the presence of other microbes, is both scientifically and practically significant. Achieving this goal requires well-designed experiments with appropriate controls, as well as careful consideration of the aforementioned factors.

A related question pertains to the regulation of the mosquito microbiome, particularly how the introduction of one microbe influences the overall composition and anti-pathogen potency of the microbiome in a given tissue. The components of the microbiome are highly variable and form a complex network. As the microbiome’s phenotypes may be outputs of the entire network, comprehensively understanding the mosquito microbiome is crucial to interpreting the findings from studies investigating the mosquito microbiome, such as inferring causality when a correlation between infection of a pathogen and differences in the microbiome is discovered. Some studies have applied a systematic and network perspective to the mosquito microbiome (Hegde et al., 2018; Ganley et al., 2020), and further network modeling may be necessary to characterize the dynamic composition of the mosquito microbiome. By approaching the mosquito microbiome as a network, more complex interactions, such as whether pathogens alter the microbiota to weaken mosquito resistance or to facilitate transmission, can be discovered, setting a theological basis for manipulating the mosquito microbiome to control mosquito-borne diseases.

Another critical question of great interest is how the mosquito microbiome is impacted by global ecological changes, such as alterations in climate and international transportation, and how these changes affect the transmission of mosquito-borne diseases. With the rapid pace of globalization, the distribution of microbes and mosquitoes has significantly changed, and urbanization and animal farming expansion have altered the frequency of mosquito-human and mosquito-animal interactions, along with the microbiomes they carry (Gubler, 2011). Climate change can also reshape the microbiota in the niches of mosquitoes, and increased temperatures alone can influence the mosquito microbiome (Jiménez-Cortés et al., 2018; de Angeli Dutra et al., 2023). These ecological factors have a profound effect on the mosquito microbiome, which in turn can modify the vectorial competence and capacity of mosquitoes. A better understanding of these interactions will help estimate the burdens of medically important mosquito-borne diseases and the risks of emergence or re-emergence of mosquito-borne pathogens.

The proposed strategies that aim to control mosquito-borne diseases by exploiting the mosquito microbiome, primarily *Wolbachia*-based techniques, have shown promising results. However, despite the encouraging results of these strategies, their application has been limited to only specific mosquito-pathogen systems and a few locations. The success of population replacement with *Wolbachia*-infected mosquitoes is particularly limited in *A. aegypti*, where naturally infecting Wolbachia is rare (Balaji et al., 2019). Introducing *Wolbachia* infection to generate pathogen-refractory lines of other mosquito species is more challenging due to interactions with highly prevalent native *Wolbachia* or mutually exclusive *Asaia* bacteria (Rossi et al., 2015). As a result, the more resource-demanding population suppression with the IIT may become the only feasible option. Even for *A*. *aegypti*, population replacement at a site requires multiple releases of *Wolbachia*-infected mosquitoes, and the sizes of releases have to be large enough that the ratio of *Wolbachia*-infected mosquitoes crosses a threshold, while being timely adjusted to the simultaneously monitored *Wolbachia* prevalence. However, despite the allocation of resources and effort, there is no guarantee that *Wolbachia* will be established due to factors such as similar populations in nearby areas and the effects of insecticides (Jiggins, 2017). Moreover, the slow spatial spread of *Wolbachia*-infected mosquitoes poses a challenge in establishing the microbe in mosquito populations outside of release sites. Ideally, a more efficient solution would be a mosquito line that can take effect after a single release of a reasonable size, or a pathogen-controlling microbe or microbial product that can be easily deployed independent of mosquitoes. To achieve the former goal, a series of CRISPR-based gene drive techniques have been proposed (Flores and O’Neill, 2018), while the latter can be achieved by probing the mosquito microbiome to identify virus- or malaria-resistant components. Another approach is paratransgenesis, which involves genetically modifying mosquito symbionts to express an effector molecule that acts against mosquito-borne pathogens (Wilke and Marrelli, 2015; Wang et al., 2022). However, these strategies come with their own technical difficulties and safety concerns. As such, the search for members of the mosquito microbiome that are efficient against pathogens and safe for application remains an ongoing pursuit.

Description: The mosquito and vertebrate microbiomes have both direct and indirect impacts on mosquito-borne disease transmission and progression, with mosquito symbiotic microorganisms showing the potential to control the diseases. Further research is needed to fully understand the interplay between mosquitoes, hosts, pathogens, and their microbiomes.

## Abbreviations

3-HK: 3-hydroxykynurenine
ADCC: antibody-dependent cellular cytotoxicity
ADE: antibody-dependent enhancement
AMP: antimicrobial peptide
APL: *Anopheles Plasmodium* responsive leucine-rich repeat protein
ATT: Attacin
BG: (1→3)-*β*-d-glucan
CD: cluster of differentiation
CEC: cecropin
CFAV: cell-fusing agent virus
cGAS: cyclic GMP-AMP synthase
CHIKV: Chikungunya virus
CI: cytoplasmic incompatibility
CTL: c-type lectin
DEF: Defensin
DENV: dengue virus
ER: endoplasmic reticulum
ERK: extracellular signal-regulated kinase
GAM: Gambicin
GI: gastrointestinal
HDAC: histone deacetylase
IFN: interferon
IgG: Immunoglobulin G
IgM: Immunoglobulin M
IIT: incompatible insect technique
IL: Interleukin
IMD: immune deficiency
iRBC: infected red blood cell
ISG: IFN-stimulated gene
ISV: insect-specific virus
JAK-STAT: Janus kinase, signal transducer and activator of transcription protein
KT: killer toxin
LACV: La Crosse virus
MAMP: microbe-associated molecular pattern
NHUV: Nhumirim virus
NK: natural killer
ONNV: o’nyong-nyong virus
PAMP: pathogen-associated molecular pattern
PDGF: platelet-derived growth factor
PGRP: peptidoglycan recognition protein
PM: peritrophic matrix
PRR: pattern recognition protein
RANTES: Regulated upon Activation, Normal T-Cell Expressed and Presumably Secreted
RELMα: resistin-like molecule-α
RNAi: RNA interference
ROS: reactive oxygen species
SFV: Semliki Forest virus
SINV: Sindbis virus
STING: stimulator of interferon genes
TLR: Toll-like receptor
TNF: tumor necrosis factor
VEC: vaginal epithelial cell
VMB: vaginal microbiome
WHO: World Health Organization
WNV: West Nile virus
YFV: yellow fever virus
ZIKV: Zika virus
